# Life Adjustment of Second‐ and Third‐Year Nurses in Japan Engaged in Shift Work Under a Health Self‐Management Program: Qualitative Analysis

**DOI:** 10.1155/jonm/9958511

**Published:** 2026-05-15

**Authors:** Akiko Nagata

**Affiliations:** ^1^ Department of Nursing, Uekusa Gakuen University, Chiba, Japan, uekusa.ac.jp

## Abstract

**Aim:**

This study aimed to interpretively explore the life adjustment processes of second‐ and third‐year nurses engaged in shift work under a health self‐management program, focusing on the integration of professional adaptation and well‐being.

**Methods:**

Seven nurses in their second or third year of clinical practice participated in a program utilizing Ryodoraku biofeedback. Data from interviews were analyzed using the Steps for Coding and Theorization method.

**Results:**

The adjustment process was conceptualized into three overarching themes: (1) navigating physiological limits and autonomous health regulation, where nurses utilized biofeedback to bridge the gap between subjective fatigue and physiological reality; (2) emotional dissonance and the struggle for agency, involving the negotiation of workplace demands; and (3) integrating professional growth and personal revitalization, where health management became a strategy for professional identity formation.

**Conclusion:**

The use of visual biofeedback served as a catalyst for participants to reflect on and transition toward proactive self‐regulation, instead of passive endurance. The findings suggest that supporting early‐career nurses requires not only rest but also tools to visualize physiological states and organizational support to foster professional agency, linking health management directly to career sustainability.

## 1. Introduction

As of late 2024, 65.7% of nurses in Japan were employed in hospitals, with approximately 110,000 (8%) early‐career practitioners being under the age of 25 [1]. Since the establishment of comprehensive nursing care standards in 1951, a three‐shift system had been the clinical norm. However, following a 1992 notification from the Ministry of Health, Labor, and Welfare (a partial revision of Health Notification No. 53, dated August 25, 1955), the adoption of two‐shift systems has rapidly expanded to accommodate various clinical needs. Consequently, two‐shift systems—often involving extended night shifts exceeding 16 hours—have become a prevalent working pattern in contemporary Japanese hospitals [2].

This situation in Japan conflicts sharply with international labor standards. While nursing in many countries follows two‐shift systems (typically 12 h shifts), shifts significantly exceeding 12 h are relatively uncommon in Western international contexts than in Japan. This trend aligns with the International Labor Organization Nursing Personnel Recommendation (No. 157) [3], which suggests that daily working time should be limited to 12 h. Empirical studies support the greater prevalence of shifts exceeding 12 h in Japan; for instance, a large‐scale study across 12 European countries reported that while shift lengths vary, 85% of nurses work 12 h or less, revealing that shifts exceeding 12 h are less frequent in most participating nations [4]. Furthermore, in the United States, although 12 h shifts are the standard, working beyond these hours is associated with increased risks to patient safety and nurse burnout, leading to organizational efforts to curb extended durations [5].

In contrast, Japan lacks labor regulations that directly restrict the duration or frequency of night shifts for individual nurses; instead, the facility standard of “average monthly night shift hours within 72 h” for medical fee reimbursement serves as the only de facto upper limit [6]. While nurses globally experience fatigue related to shift work [5], the severity and nature of this fatigue are compounded by shift duration. Specifically, the 16‐h shifts prevalent in Japan have been associated with higher levels of acute exhaustion and slower recovery compared to shorter shifts [7]. Japanese nurses frequently report severe subjective symptoms such as chronic fatigue, malaise, and persistent drowsiness, which are exacerbated by these exceptionally long shifts. Despite these enduring challenges, fundamental measures to alleviate the burden of such intensive long‐hour shifts remain insufficient.

While addressing these organizational and systemic challenges is critical, understanding the individual adaptation processes through which nurses navigate their demanding daily shifts and sustain their careers is equally important. In particular, second‐ and third‐year nurses—who have progressed beyond their initial orientation year—face increased clinical responsibilities and high physical and mental strain. This phase represents a critical transition for developing professional identity and building resilience. However, while previous literature has extensively addressed organizational interventions, the proactive processes through which nurses manage their health (self‐management) and adjust their daily lives to adapt professionally remain lacking.

Therefore, this study aimed to interpretively explore the subjective life adjustment processes of second‐ and third‐year nurses engaged in shift work under a health self‐management program, focusing on their professional adaptation and growth.

## 2. Aim and Operational Definition

### 2.1. The Study Aim

This study interpretively explored the individual adjustment processes of second‐ and third‐year nurses engaged in shift work who participated in a health self‐management program. Beyond mere description, this study sought to elucidate how these early‐career nurses navigate the physiological and psychological challenges of intensive shift work to achieve professional adaptation. By identifying the mechanisms of their life adjustment, we aimed to provide practical recommendations for nursing management to enhance nurse retention and well‐being.

### 2.2. Operational Definition of Life Adjustment

For this study, “life adjustment” was defined as a proactive and reflective endeavor in which individuals recognize, accept, and regulate their physical and mental states—including beliefs, emotions, and intentions. This process is viewed as a foundational element of professional identity formation and resilience, where personal health management and professional life mutually influence one’s overall adaptation to the clinical environment.

## 3. Materials and Methods

### 3.1. Design and Theoretical Framework

We employed a qualitative descriptive‐analytical design to explore the subjective efforts of nurses in managing their health and adapting to shift work. This design is appropriate for elaborating on participants’ personal views and their nuanced understanding of specific life experiences [8]. The study is theoretically grounded in the concept of self‐regulation [9, 10] and professional identity development [10–13]. This focused framework allowed us to examine how nurses cognitively and behaviorally regulate their health in response to work environment stressors and how these self‐regulatory processes are intertwined with their emerging professional identities.

### 3.2. Study Setting and Recruitment

#### 3.2.1. Eligibility

Participants were female nurses in their second or third year of clinical practice, employed at general hospitals providing highly advanced medical care. Eligibility required participants to be cognizant of shift‐work‐related health challenges (e.g., fatigue, menstrual irregularities, and musculoskeletal pain) and to have a proactive desire to improve these conditions through self‐management.

#### 3.2.2. Sampling Method

Snowball sampling was utilized. Initial contact was made with a nurse at a tertiary care hospital, who then referred subsequent participants meeting the inclusion criteria. Seven participants were invited, and all agreed to participate (100% recruitment rate).

#### 3.2.3. Determination of Sample Size

Data were collected and analyzed concurrently using a constant comparative approach to ensure validity.

Recruitment was discontinued at seven participants. While we acknowledge that theoretical saturation is difficult to claim with a small, homogenous sample, we determined that sufficient information power [14] was achieved for this study. According to Malterud et al., a smaller sample size is justified when the information power is high. In this study, the information power was considered high because (1) the study aim was narrowly focused on a specific adjustment process; (2) the sample was highly specific (second‐ and third‐year shift‐working nurses in Japan); (3) the analysis was supported by established theories (self‐regulation and professional identity); (4) the dialogue quality was strong, as the Ryodoraku [15] biofeedback served as a concrete catalyst for deep reflection; and (5) the analysis strategy (Steps for Coding and Theorization [SCAT] method [16]) focused on an in‐depth exploration of narratives rather than cross‐case variation. Therefore, we deemed a sample of seven participants as appropriate for fulfilling the exploratory aim of this qualitative inquiry.

#### 3.2.4. Inclusion and Exclusion Criteria

Inclusion required full participation in pre‐ and postprogram interviews and all eight in‐program sessions. This ensured the longitudinal depth of the data required for SCAT analysis [16].

### 3.3. Data Collection: The Health Self‐Management Program

The program was conducted over three months (April 2013 to December 2014). It was designed to foster self‐regulation through the following six core pillars:

1. Heightening curiosity regarding dietary habits through continuous records.
2. Linking practical nutritional knowledge to behavioral modifications.
3. Prioritizing personal acknowledgment as a precursor to adjustment skills.
4. Fostering awareness of diverse bodily functions.
5. Identifying individual directions for adjustment through mindfulness of positive experiences.
6. Cultivating cognitive patterns to recognize physiological fluctuations and consistencies.

#### 3.3.1. Implementation and Ryodoraku Biofeedback

Participants self‐monitored their health status (e.g., basal body temperature, sleep‐wake cycles, and subjective symptoms) and received biweekly coaching. At each session, the researcher utilized Ryodoraku to provide objective biofeedback. By measuring electrical skin resistance, Ryodoraku visualized the functional state of the participants’ autonomic nervous system. This visual data (printed results of sympathetic excitation levels) served as a “reflective mirror,” enabling nurses to correlate their subjective fatigue with objective physiological markers, thereby facilitating more precise life adjustments.

#### 3.3.2. The Self‐Management Program and Ryodoraku

As part of the health self‐management program, we utilized the Ryodoraku tool to provide participants with objective biofeedback. Ryodoraku measures electrical skin resistance at specific points, reflecting the functional state of the autonomic nervous system. We selected this approach because it allows nurses to visually monitor their physiological stress levels, facilitating a more reflective self‐regulation process in their daily lives. By integrating Ryodoraku data with qualitative interviews, we aimed to capture a holistic view of how nurses balance physiological recovery and professional adaptation.

It is important to note that while Ryodoraku provides numerical values representing electrical skin resistance, it is a complementary tool rooted in Eastern medicine rather than a definitive biomedical diagnostic instrument. In this study, its primary function was not to medically diagnose participants but to serve as a visual, contextual tool that prompted nurses to reflect on their internal states and initiate a meaning‐making process regarding their fatigue.

### 3.4. Data Analysis

#### 3.4.1. Individual Analysis Process

All interviews were recorded with the participants’ informed consent. To ensure anonymity, verbatim transcripts were created with all personally identifiable information removed. The analysis adhered to the following rigorous four‐step coding process to transition from raw data to conceptual themes:

• Step 1: Focused text extraction. Key expressions relevant to the nurses’ life management and experiences with shift work were extracted.
• Step 2: Paraphrasing. Participants’ vernacular was replaced with more general expressions while preserving the original nuances.
• Step 3: Conceptualization (explanatory terms). The paraphrased data were linked to academic concepts derived from the study’s theoretical framework, such as “self‐regulation” [12] and “environmental adaptation” [17]. Specifically, participants’ internal states and behavioral changes were encoded using terms like “biofeedback‐driven awareness” and “professional role conflict,” allowing for a theoretical interpretation of how nurses maintain homeostasis within their demanding work environment.
• Step 4: Integration (themes and structural concepts). These concepts were synthesized into overarching themes that describe the essential structure of the nurses’ adaptation process.

To ensure the rigor and transparency of the analysis, the constant comparative method was employed. The primary researcher conducted the initial coding, after which co‐researchers reviewed the content to enhance the credibility of the findings. Specific examples of the coding process using SCAT are presented in Table 1.

**TABLE 1:** Part of the form for analyzing verbatim transcriptions of utterances using SCAT.

Step 1: Focused text extraction	Step 2: Paraphrasing	Step 3: Conceptualization (explanatory terms)	Step 4: Integration (themes and structural concepts)
“Seeing my condition reflected as Ryodoraku data allowed me to visually understand my state. Moreover, receiving advice, such as what I should eat, was helpful—it was different from just brooding alone about being in poor health.”	Transitioning from passive endurance of fatigue to objective recognition of physical status through data.	Biofeedback‐driven awareness: bridging the gap between subjective and objective fatigue (self‐regulation).	Shift from passive reaction to autonomous health management.
“I think I’ve started to notice things. For example, after a night shift, I realize the fatigue in my legs is due to stiffness in this specific area. Before, I just thought everyone feels heavy after a shift, but now I specifically think about massaging where it’s stiff.”	Implementing specific environmental and behavioral adjustments to maintain physical balance.	Proactive behavioral adjustment: physical self‐regulation (self‐regulation theory).	Integration of professional roles and personal well‐being.
“Being healthy allows me to care for patients with confidence, even during busy night shifts.”	Realizing that self‐health management directly enhances professional performance and confidence.	Professional self‐efficacy: synergy between self‐care and professional identity.	Cultivating resilience through self‐regulation.

#### 3.4.2. Overall Analysis

We scrutinized the commonalities, parallels, and distinctions in the “attributes of professional and personal life” across all participants to elucidate the overarching dynamics of nurses’ lives within shift‐work schedules. Lifestyle characteristics were analyzed through the lens of holistic well‐being, viewing participants as integrated human entities. Through this process, we unraveled the underlying structures of their daily routines and identified the nuanced adjustments made by second‐ and third‐year nurses.

#### 3.4.3. Ensuring Credibility and Rigor

Throughout data collection and analysis, the researchers assessed the suitability of the methods employed and the validity of the findings by cross‐referencing and constantly comparing the data. To ensure rigor and reflexivity, the researchers provided participants with a summary of the previous interview at the start of each session for validation (member checking). The primary researcher, belonging to a different organization than the participants to ensure a free and open dialogue, maintained an independent stance. The researcher possesses 14 years of experience as a facilitator in nursing case study meetings and holds significant expertise in assessing health status and guiding nursing care. Furthermore, the researcher received specialized training in Ryodoraku measurement and data interpretation from a member of the Japanese Ryodoraku Autonomic Nervous System Society. This expertise was further applied over 4 years of teaching health self‐management to nursing students. Coupled with 4 years of clinical experience in a three‐shift system at an advanced medical institution, the researcher possesses the necessary professional background to ensure the depth and accuracy of the analysis.

Furthermore, we actively engaged in reflexivity regarding the primary researcher’s dual role as both the program facilitator and interviewer. We recognized the inherent power dynamics and risk of social desirability bias—where participants might overstate the program’s benefits—as well as the potential for confirmation bias during analysis. To mitigate these risks, the analytical process relied heavily on verbatim transcripts rather than the researcher’s subjective impressions. We also employed peer debriefing; the coding and analytical interpretations were critically reviewed by independent nursing research colleagues to provide an objective perspective and challenge solitary researcher bias. Additionally, continuous dialogue with participants during the program—where Ryodoraku data, dietary records, and subjective physical conditions were shared and verified in real‐time—ensured that our foundational understanding remained closely tethered to the participants’ actual, immediate lived experiences.

### 3.5. Ethical Considerations

This study was approved by the Ethics Review Board of the Chiba University Graduate School of Nursing (Approval No. 24‐40; Date: October 22, 2012). Participants were provided with written and verbal explanations regarding the research aims, their expected role, potential burdens, the voluntary nature of participation, and their right to withdraw at any time without repercussions.

Throughout the study, participants’ autonomy was prioritized. We ensured their physical and psychological comfort, avoided any risks or disadvantages, and strictly safeguarded their privacy, anonymity, and personal information. To minimize the burden, interviews were conducted after participants’ shifts, only after confirming that they were in a suitable physical and mental condition to engage in the session. All research activities were conducted in accordance with ethical principles prioritizing the participants’ rights and well‐being.

## 4. Results

### 4.1. Study Population

The characteristics and health statuses of the seven participants before commencing the program are outlined in Table 2.

**TABLE 2:** Program participants.

	A	B	C	D	E	F	G
Age	23	23	24	24	22	24	23

Basic education	4‐year university	4‐year university	4‐year university	4‐year university	Vocational school	4‐year university	4‐year university

Clinical experience	2nd year	2nd year	3rd year	3rd year	2nd year	2nd year	2nd year

Ward	Esophageal/gastrointestinal surgery	Respiratory Surgery/Internal Medicine	Orthopedic Surgery	Orthopedic Surgery	Gastroenterology	Gastroenterology	Gastroenterology

Living situation	Living alone (dormitory)	Living alone (dormitory)	Living alone	Living alone (dormitory)	Living alone (dormitory)	Living alone (dormitory)	Living alone
			(apartment)				(apartment)

Motivation to participate	Upon commencing my employment, I strongly felt the necessity to prioritize my health and well‐being	Night shifts are very draining for me	I was invited	I was invited	I was invited	It seemed appealing during the orientation	I was invited
		I think my food habits are very disordered; I want to reconsider my lifestyle	I am interested in Eastern medicine	I am interested in research activities	I wanted a yoga mat	I wanted a yoga mat. I have done yoga before and found it enjoyable	I am interested in my health
			My health isn’t good, and I need to fix it				

Health problems	Atopic dermatitis	Shoulder pain (since college)	Easily tired	Gained weight	Constipation, lower back pain, and shoulder pain	My menstrual pains have gotten unbearable	My skin is not in good shape
	Chronic fatigue, shoulder pain,	Irregular menses due to stress	Very tired after day shifts, even if not busy	Snoring has become a problem	Worried about what other people think of me		Irregular menstruation (related to work stress)
	lethargic, unable to sleep	Also have menstrual pain and take painkillers	No motivation	Breathing through my mouth	I like Japanese sweets, and I eat too many of them		Shoulder pain, lower back pain (since high school)
		Often get irregular bowel movements	Lower back pain				

Health management	Supplements	Gym biweekly (yoga/swimming)	Running	Running at the gym (1–2 times per week)	I eat veggies	I want to have a healthier body, so I’ve started going to the gym	I don’t eat oily foods
	Massage/chiropractor			I eat breakfast	(Lettuce, tomato, cucumber)		I eat veggies and yogurt
	I don’t eat cup noodles			Vitamin supplements			I eat breakfast
							I do yoga at the gym (once a week)

### 4.2. Conceptual Themes of Life Adjustment in Early‐Career Nurses

Through the SCAT analysis, 13 specific categories were identified and synthesized into three overarching themes that characterize the subjective adjustment process of second‐ and third‐year nurses. These themes illustrate the dynamic interplay between physiological regulation, psychological agency, and professional identity formation. Table 3 summarizes this synthesis process from subcategories to overarching themes.

**TABLE 3:** Correspondence between the 13 subcategories and the 3 overarching themes.

Overarching themes	Subcategories (original 13 categories)	Conceptual integration and definition
Theme 1: Navigating Physiological Limits and Autonomous Health Regulation	1. Awareness of physical changes due to night shifts and continuous work 2. Awareness of changes in daily life rhythms and strain due to working conditions 3. Continuous, intensive work leading to fatigue 10. Autonomous improvement of eating habits through objective understanding of diet‐health links 11. Habituation of lifestyle adjustments through physical awareness and sensory pleasure 12. Disruption in adjustment due to lack of physical improvement or difficulty in tool utilization	Describes the process of moving from passive endurance of shift‐work stressors to active self‐regulation. It highlights how objective biofeedback (e.g., Ryodoraku) helps nurses bridge the gap between subjective fatigue and physiological reality.

Theme 2: Emotional Dissonance and the Struggle for Agency in the Workplace	4. Regular overtime stemming from responsibility for the nursing process 5. Perceived sense of control over job performance and work environment 6. Constant tension in performing work duties 9. Existence of a working environment that guarantees growth and security	Focuses on the psychological struggle to maintain professional agency (a sense of control). It illustrates the tension between expanding responsibilities and the need for a supportive organizational climate to sustain mental and physical health.

Theme 3: Integrating Professional Growth and Personal Revitalization	7. Relieving professional tension through private‐life activities: Revitalization and fatigue 8. Recognizing and tackling issues to improve nursing practice abilities 13. Reviewing the connection between Ryodoraku results, daily activities, and holistic states	Represents the synthesis of the “professional self” and “personal self” into a holistic entity. It shows how the drive for clinical excellence motivates health self‐management, aiming for sustainable harmony between career and life.

Theme 1, “Navigating Physiological Limits and Autonomous Health Regulation,” was derived from six subcategories (1, 2, 3, 10, 11, and 12) focusing on physical awareness and the transition to proactive self‐care. Theme 2, “Emotional Dissonance and the Struggle for Agency in the Workplace,” consolidated four subcategories (4, 5, 6, and 9) related to work‐related stress and the influence of the organizational environment. Finally, Theme 3, “Integrating Professional Growth and Personal Revitalization,” integrated three subcategories (7, 8, and 13) that describe the holistic synthesis of professional identity and personal well‐being.

### 4.3. Theme 1: Navigating Physiological Limits and Autonomous Health Regulation

The participants demonstrated an acute awareness of physical deterioration—such as edema, chronic fatigue, and sleep‐wake cycle disruptions—caused by the 16‐h shift‐work environment. Initially, their response to these symptoms was reactive or transient. However, through the self‐management program, a shift occurred from passive endurance to autonomous regulation. The use of objective biofeedback via Ryodoraku and meal records served as a “reflective mirror,” allowing participants to bridge the gap between their subjective exhaustion and objective physiological data. This awareness fostered proactive dietary improvements and the adoption of self‐care habits (e.g., deep breathing and specific bathing routines). Conversely, when physiological improvements were not immediately visible, participants struggled with habit formation, highlighting the critical role of continuous, visible feedback in sustaining health‐seeking behaviors.

### 4.4. Theme 2: Emotional Dissonance and the Struggle for Agency in the Workplace

This theme captures the psychological tension between growing clinical responsibilities and the perceived sense of control (agency). Second‐ and third‐year nurses frequently faced role strain due to heavy workloads. A critical finding was the dichotomy between stability and instability: organizational support and a clear sense of control enhanced resilience, whereas being assigned roles beyond their developmental stage (e.g., night shift leader) without adequate support led to anxiety and resentment.

### 4.5. Theme 3: Integrating Professional Growth and Personal Revitalization

**FIGURE 1:**
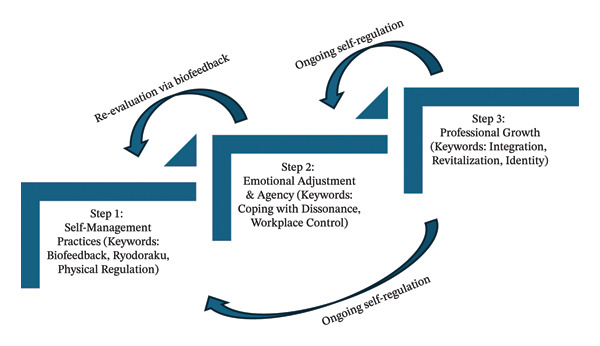
The Conceptual Model of Life Adjustment in Early‐Career Nurses. *Note.* This model illustrates the hierarchical relationship between the three themes. (1) Foundation: Objective biofeedback facilitates “Autonomous Health Regulation,” serving as the physiological basis. (2) Process: This regulation empowers nurses to navigate “Emotional Dissonance” and establish agency within the workplace. (3) Outcome: Ultimately, these efforts are integrated into “Professional Growth,” where health management becomes a proactive strategy for sustainable nursing practice.

The adjustment process encompassed the integration of the “professional self” and the “personal self.” While private‐life activities provided vital revitalization, the pursuit of nursing practice excellence served as the primary intrinsic motivator for health management. By synthesizing Ryodoraku results with daily activities, participants began to perceive themselves as integrated human entities, seeking sustainable harmony between clinical career growth and holistic well‐being (Figure 1).

## 5. Discussion

The findings of this study provide a theoretical depiction of the complex adjustment processes undertaken by second‐ and third‐year nurses working intensive 16 h shifts. The integration of Ryodoraku biofeedback with a health self‐management program revealed that individual adaptation is not merely a matter of physical rest but a dynamic process of developing self‐regulation and professional agency.

### 5.1. The Role of Biofeedback in Self‐Regulation

As identified in Theme 1, the transition from passive fatigue to autonomous health regulation was facilitated by objective biofeedback. In nursing management, the “visibility” of physiological strain is often overlooked. Our results suggest that tools like Ryodoraku act as a “reflective mirror.” In other words, regardless of the debates surrounding its absolute biomedical validity, the visualized data played a crucial psychological role for the participants. It bridged the gap between a nurse’s subjective perception of “being fine” and the physiological reality of autonomic imbalance. This aligns with self‐regulation theory, which posits that objective feedback is a prerequisite for behavioral modification [12]. For nursing managers, providing such reflective tools could be a proactive strategy to prevent burnout before subjective symptoms become debilitating.

### 5.2. Professional Agency and Organizational Support

Theme 2 highlighted the struggle for agency within the workplace. The 2^nd^ and 3^rd^ years of nursing are a “critical transition” where role strain increases as clinical responsibilities expand [11]. Our findings indicate that a nurse’s ability to manage their health is inextricably linked to their perceived sense of control over their work environment [13]. When nurses felt unsupported or overwhelmed by roles beyond their developmental stage (e.g., night shift leader), their self‐management efforts were disrupted. This finding underscores the necessity for nursing administrators to ensure that workload distribution and team composition are balanced not just for patient safety but also for the developmental sustainability of the nursing workforce.

### 5.3. Professional Identity and Holistic Well‐Being

Finally, Theme 3 illustrates that professional growth and personal life are not separate entities but are integrated through a search for harmony. The pursuit of “nursing practice excellence” (Theme 3) served as a powerful intrinsic motivator for health management. This result suggests that in the early‐career stages, professional identity formation and resilience‐building are mutually reinforcing [10, 18]. Nursing management should therefore view health programs not as isolated “wellness initiatives,” but as an integral part of professional development and career‐long retention strategies.

Furthermore, managing fatigue in shift work is not merely a biological challenge but a critical component of self‐regulation necessary for nursing sustainability [19]. Previous studies have highlighted that unmanaged work‐related strain significantly increases burnout and turnover intentions among early‐career nurses [20]. In particular, recent research emphasizes that fostering individual resilience and psychological well‐being is paramount for improving the retention of new and early‐career nurses in the post‐pandemic era [21]. My findings suggest that the Ryodoraku‐based biofeedback program serves as a practical intervention tool to support this well‐being. Visualizing their physiological status empowers nurses to engage in autonomous health management, which may ultimately mitigate burnout and enhance the sustainability of the nursing workforce in shift‐based environments.

While previous qualitative studies have extensively documented the stress and transition shock experienced by early‐career nurses, this study offers a novel conceptual contribution by elucidating how self‐regulation is achieved under extreme conditions, such as working 16‐h shifts. The findings suggest that providing tools to visualize invisible physiological strain does more than merely promote physical rest; it disrupts the cycle of “passive endurance” that often leads to burnout. By objectifying their fatigue, nurses are empowered to reclaim their professional agency. Therefore, the unique insight of this study is that physiological self‐regulation, triggered by visual feedback, is not separate from, but rather a fundamental prerequisite for, the psychological integration of professional identity in highly demanding clinical environments.

### 5.4. Limitations

This study has some limitations. First, the small sample size (*N* = 7) and the use of snowball sampling within a specific clinical context limit the transferability of the findings. Second, as a qualitative exploration, this study does not establish the causal efficacy of the self‐management program or the clinical validity of the Ryodoraku tool. Future research employing mixed methods or longitudinal designs with larger cohorts is needed to evaluate the impact of such interventions on burnout and retention quantitatively.

## 6. Conclusions

This study elucidated the health regulation process of nurses engaged in shift work through objective autonomic nerve measurements using Ryodoraku and interviews. The analysis conceptualized the nurses’ experiences into three major phases: “Navigating Physiological Limits and Autonomous Health Regulation,” “Emotional Dissonance and the Struggle for Agency in the Workplace,” and “Integrating Professional Growth and Personal Revitalization.”

Initially, nurses tended to passively accept the physical toll of night shifts and excessive workloads. However, by confirming objective numerical values (biofeedback) provided by Ryodoraku, they recognized the discrepancy between their subjective fatigue and physiological reality. In our interpretative analysis, this recognition appeared to be a crucial turning point, prompting participants to attempt autonomous lifestyle regulation.

However, this process of self‐regulation can be hindered by a lack of perceived control in the workplace environment and the dissonance associated with emotional labor. The results of this study suggest that the most effective adaptation occurs when nurses overcome these barriers and reframe health management not merely as an “obligation,” but as a means to integrate “maintaining professional performance (professional growth)” with “enriching personal life (personal revitalization).”

Therefore, organizational support should not be limited to merely encouraging rest. Providing tools that allow nurses to objectively view their physiological state and fostering an environment where they can exercise professional autonomy are crucial for the sustainable continuation of their careers.

## Funding

This research received no specific grant from any funding agency in the public, commercial, or not‐for‐profit sectors.

## Disclosure

The final manuscript was thoroughly reviewed and validated by the author who assumes full responsibility for the integrity and accuracy of the work.

## Ethics Statement

The study was approved by the ethics review board at Chiba University Graduate School of Nursing (approval number: 24‐40). All study participants provided informed consent.

## Conflicts of Interest

The author declares no conflicts of interest.

## Data Availability

The data are available from the author upon request.
